# Functional characterization of an aquaporin from a microsporidium, *Nosema bombycis*

**DOI:** 10.1371/journal.pone.0181703

**Published:** 2017-07-27

**Authors:** Gong Chen, Wei Wang, Hongli Chen, Weijiang Dai, Xiangran Peng, Xiaoliang Li, Xudong Tang, Li Xu, Zhongyuan Shen

**Affiliations:** 1 Jiangsu University of Science and Technology, Zhenjiang, Jiangsu Province, China; 2 Sericultural Research Institute, Chinese Academy of Agricultural Sciences, Zhenjiang, Jiangsu Province, China; 3 Key Laboratory of Genetic Improvement of Silkworm and Mulberry of Agricultural Ministry, Zhenjiang, Jiangsu Province, China; Weizmann Institute of Science, ISRAEL

## Abstract

Microsporidia are a diverse group of eukaryotic organisms, capable of causing parasitic infections in both vertebrates and invertebrates. During the germination process, there is an increase in the osmotic pressure of microsporidian spores. As part of this study, we cloned a homologous aquaporin gene in *Nosema bombycis*, and named it *Nosema bombycis* aquaporin (*NbAQP)*. Sequence analysis revealed that the *NbAQP* contains an open reading frame with a length of 750 bp and encodes a polypeptide of 249 amino acids. Amino acid sequence homology was greater than 50% that of five aquaporins from other microsporidian species. Indirect immunofluorescence (IFA) and immunogold electron microscopy showed NbAQP to be located predominantly in the spore wall of *N*. *bombycis* spores. The results of qRT-PCR analysis revealed that *NbAQP* expression remained high 0 h after inoculation and decreased sharply to 24 h, increased gradually from 2 days and peaked at 6 days. After expression of NbAQP in *Xenopus laevis* oocytes, it was observed that NbAQP can promote rapid penetration of water into oocytes. The associated permeation rate was 2–3 times that of the water-injected and uninjected oocytes. Antibody blocking experiments showed that the inhibition rate of spore germination was approximately 28% after antibody blocking. The difference in germination rate between the control group and the NbAQP group was significant (*P* < 0.05). This study shows for the first time that *N*. *bombycis* contains functional water channel proteins and provides a platform suitable for further research into the mechanisms underlying the regulation of NbAQP protein expression. Further study of NbAQP and their inhibitors may have significance for prevention of microsporidiosis.

## Introduction

Microsporidia are unicellular eukaryotic organisms capable of causing parasitic infections in both vertebrates and invertebrates. Silkworm pebrine disease, caused by *N*. *bombycis*, cause great losses in sericulture all over the world [1]. Furthermore, although a lot of progress has been made in relation to research associated with *N*. *bombycis* infection, prevention and control [2], the mechanisms underlying infection remain to be elucidated. Microsporidian spore germination is an important process during host infection [3]. It is commonly believed that spore germination and infection progress through 5 stages: (1) spore adhesion to host cells; (2) spore activation; (3) increases in the internal osmotic pressure of the spore; (4) polar filament ejection; and (5) sporoplasm injection into the cytoplasm of the host cell through the polar filament. Research has revealed that EnP1 from *Encephalitozoon cuniculi* [4] and a spore wall protein (SWP26) [5] of *N*. *bombycis* are involved in adhesion of microsporidia to the host. The mechanisms for the germination of microsporidian spore and dynamics of polar filament discharge and sporoplasm expulsion have also been reported in previous studies [4–8]. However, the specific mechanisms that underlie these reactions, especially the activation and increases in the internal pressure of the spore, remain to be elucidated.

The aquatic channel protein belongs to the MIP superfamily and is capable of promoting water and solute (small molecules) transport. These proteins have been observed in bacteria and humans, and they are especially prevalent in plants. Early research has suggested that aquaporins are only capable of transporting water molecules in several mammalian species. However, many subsequent studies have revealed that members of the aquaporin family are capable of transporting a wide variety of solutes including glycerol, urea, carbon dioxide, and nitric oxide [9, 10]. During germination, there is an increase in the osmotic pressure of microsporidian spores. The prevailing belief is that transportation of water from the outer environment into the spores and/or metabolism of sugar may play an important role [11]. Nevertheless, this hypothesis has not been proved adequately through broad research. Although a putative aquaporin was characterized in the microsporidium *Encephalitozoon cuniculi* in 2006 [12], more reports about aquaporins of other microsporidia and its specific function have not emerged. The current study was conducted to investigate the characteristics and biological function of the aquatic channel protein in the microsporidium *N*. *bombycis*. This study may provide a platform for future research into the mechanism underlying the germination and prevention of pebrine diseases in silkworm.

*Xenopus oocytes* detection system is the method most commonly used for aquaporin. As the success of many ion channel gene cloning, *Xenopus laevis* oocytes were widely used as heterologous ion channel expression vectors. The study of the relationship between the structure and function of ion channels become possible through the combination of molecular biology and electrophysiological methods [13]. The expression of exogenous ion channel genes by *Xenopus laevis* oocytes is an important method in the study of the structure and function of ion channels. This system is convenient because the endogenous water permeability coefficient (Pf) of the oocyte membrane is low and water transport into the oocyte can be measured with high accuracy [14].

## Materials and methods

### Preparation of *N*. *bombycis* spores and *Xenopus laevis* oocytes

The silkworm p50 strain and *N*. *bombycis* spores were obtained from the Sericulture Research Institute of the Chinese Academy of Agricultural Sciences (http://dx.doi.org/10.17504/protocols.io.if3cbqn). Preparation of *Xenopus laevis* oocytes: *Xenopus laevis* were put in ice water until unconscious (without any reaction to a leg pinch), then the ovary lobes were carefully dissected and washed 4 times in a Petri dish (50 mm) with ND96 medium (96 mM NaCl, 2 mM KCl, 1 mM MgCl_2_, 1.8 mM CaCl_2_, 5 mM HEPES adjusted to pH 7.4 with NaOH, supplemented with 50 μg/ml streptomycin/penicillin, and 225 μg/ml sodium pyruvate) to remove all traces of blood and debris. The wounded body wall and skin of *Xenopus laevis* were closed with a 3–0 silk suture on a 24 mm curved needle. The female was allowed to recover in an aquarium 1 g/L salt in water. The isolated *Xenopus laevis* oocytes were digested with 2 mg/mL of collagenase A (Sigma^TM^) without Ca^2 +^ modified Barth’s solution (88 mM NaCl, 1 mM KCl, 2.4 mM NaHCO_3_, 10 mM Hepes-NaOH, 0.82 mM MgSO_4_, pH 7.4, 200 mOsm/kg) at room temperature for 2 h [14].

### Ethics statement

This study was performed in strict accordance with the recommendations in the Guide for the Care and Use of Laboratory Animals of the National Institutes of Health. The protocol was approved by the Laboratory Animal Management Committee of Jiangsu University (Permit Number: UJS-LAER-2016120201). All surgery was performed under *Xenopus laevis* unconscious, and every effort was made to minimize suffering.

### Cloning and sequence analysis

The *NbAQP* gene in *N*. *bombycis* was identified using the MicrosporidiaDB (http://microsporidiadb.org/micro/) and MIPModDB (http://bioinfo.iitk.ac.in/MIPModDB/index.html) databases. The following PCR primers were designed based on homologous gene sequences: upstream primer, 5’- ATGACCAGAGAGACATTGAAG -3’ and downstream primer, 5’- CTAAAAGCTGAGCTTGTACAG -3’. Genomic DNA was extracted from *N*. *bombycis* spores by Dong et al. (https://dx.doi.org/10.17504/protocols.io.igacbse) [15]. The resultant DNA was used as a template to amplify *NbAQP*. The associated amplicons were sequenced by Sangon Biotech (Shanghai) Co., Ltd. The basic physical and chemical properties of the signal peptide and the transmember domain were predicted using SignalP 4.1 (http://www.cbs.dtu.dk/services/SignalP/), Inter Pro (http://www.ebi.ac.uk/interpro/), TMpred (http://www.ch.embnet.org/software/TMPRED_form.html), and Expasy (http://web.expasy.org/compute_pi/). This analysis also facilitated prediction of the molecular mass and isoelectric point of the protein encoded by the *NbAQP* gene. The tertiary structure of NbAQP was predicted using SWISS-MODEL (https://swissmodel.expasy.org/). The six homologous sequences including NbAQP were compared using Clustal X software. The evolutionary tree of AQPs, including NbAQP, was generated using MEGA6.

### In vitro expression of NbAQP and preparation of antibodies

*NbAQP* was cloned into a pYES2-NTC vectorusing *Kpn*I and *Xho*I restriction sites. NbAQP was amplified by PCR using 100 ng of the template, which isolated from *N*. *bombycis*. PCR was performed using 25μl of PrimeSTAR HS DNA Polymerase (Takara Biotechnology (Dalian) Co., Ltd.) in a final volume of 50μl (denaturing temperature 95°C for 5 min, annealing temperature of 55°C for 30 s, and extension temperature of 72°C for 1 min; 30 cycles) and 2 μl of each primer. The PCR primers used were as the following sequences: F: 5′-CGGGGTACCATGGTATCAAGAAATATATT-3′ (the underlined area is the *Kpn*Ⅰ restriction site); R: 5′-CCGCTCGAGTTAATAAAGCTTGTACAATATCG-3′ (the underlined area is the *Xho*Ⅰ restriction site). The fusion protein containing the N-terminal His tag was determined to be approximately 31 kDa. pYES2-NTC-NbAQP was subsequently transformed into the *S*. *cerevisiae* strain, INVSc1 and induced with β-galactose. Then, the induced cells were collected by centrifugation at 15 h, 20 h, and 24 h after induction. The cells were washed with precooled PBS buffer (pH 7.4) and resuspended in precooled lysate. The expression of the fusion protein was detected using 12% SDS-PAGE. If the fusion protein was in the inclusion body, the precipitate was centrifuged and dissolved overnight at 4°C with 8 M urea solution. If the fusion protein was in the supernatant, the cells were disrupted with ultrasound and the supernatant was collected by centrifugation. Protein purification was performed according to the instructions outlined by manufacturer of the His Trap FF affinity chromatography column (GE Healthcare^TM^). SDS-PAGE and Western blotting were used to identify the extracted protein, using His-tagged mouse antibody (Sangon Biotech (Shanghai) Co., Ltd.) as a primary antibody and HRP-labeled goat anti-mouse antibody (Sangon Biotech (Shanghai) Co., Ltd.) as a secondary antibody.

The antigen protein was mixed with phosphate buffered saline (PBS). Then antigen solution were injected into Freund’s adjuvant, which was prepared by warming in a beaker of warm water. Finally, the mixture was vortexed vigorously for 2 min to produce a good suspension. Each rabbit was subcutaneously injected with 0.5 mg protein. Injections were performed every 4–6 weeks, with bleeds 7–10 days after each injection. After antiserum antigen affinity purification of antibodies, the purified antibody was obtained and the quality of the antibodies in serum of the bleeds was monitored by indirect ELISA. Preimmune serum was taken as negative control.

### Localization of NbAQP in the *N*. *Bombycis* spore

The purified spores were fixed in 3–4% paraformaldehyde in PBS pH 7.4 for 15 min at room temperature. The spores were washed twice with ice cold PBS, then incubated for 10 min with PBS containing 0.25% Triton X-100. After washing in PBS, these spores were incubated for 1 h with PBS containing 1% BSA at room temperature. The spores from the experimental group were subsequently incubated with the prepared polyclonal antibody (1:50) for 1 h, while the control group was incubated with negative serum for 1 h. Following incubation, the spores were centrifuged for 15 min at 8000 rpm prior to removal of the supernatant. Fresh PBS buffer containing 1% BSA was added and the resultant mixture was incubated with HRP-labeled mouse anti-rabbit secondary antibody (Sangon Biotech (Shanghai) Co., Ltd.) (1:50) for 1 h. The mixture was once more centrifuged for 20 min at 8000 rpm, and the supernatant was removed. Then 200 ul PBS + 0.1% Triton X-100 (PBST) and RNase (at a final concentration of 100 μg/ml) were added to the eluate and the mixture was incubated at room temperature for 4 h. A 3 μM Propidium Iodide (PI) stain solution was added and incubated at room temperature for 20 min. The samples were collected by centrifugation for 15 min at 8000 rpm and washed with PBST. Photographs were taken of the samples following visualization using a laser confocal microscope (Leica SP8, Germany).

A revised version of a previously published Immunogold electron microscopy method was used to visualize the NbAQP protein [16]. Briefly, the *N*. *bombycis* samples were adsorbed on a nickel mesh. The samples were subsequently blocked for 20 min using blocking solution (0.8 g Albumin, bovine, fraction V, heat shock isolation pH 7.0 solve in 20 mL 1× PBST). Next, the diluted polyclonal antibody (primary antibody) was added for 2 h and the samples were subsequently washed three times with PBS. The diluted gold standard anti-rabbit secondary antibody (Sangon Biotech (Shanghai) Co., Ltd.) was then added and the samples were washed three times with PBS. Next, the samples were fixed with 0.5% glutaraldehyde and rinsed three times with double-distilled water. Uranyl acetate was subsequently added and the mixture was incubated for a further 3 min prior to washing (several times) with double-distilled water. Next, lead citrate was added to the mixture and incubated for 3 min prior to washing (several times) with double-distilled water. Finally, the samples were rinsed with 0.04% Triton X-100, dried at room temperature, and observed using transmission electron microscopy.

### Real-time fluorescence quantitative PCR

Real-time quantitative PCR was performed using the SYBR Premix Ex Taq II kit and an Applied Biosystems 7300 Real-Time PCR System. cDNA generated from genomic DNA extracted from *N*. *bombycis* served as a template. The *β-tubulin* gene of the *N*. *bombycis* served as the reference gene [17]. It was amplified using specific primers: 5′-TTCCCTTCCCTAGACTTCACTTC-3′ (F) and 5′-CAGCAGCCACAGTCAAATACC-3′ (R). The length of the amplified *NbAQP* fragment was predicted to be 174 bp. The upstream primer sequence used to amplify the target gene was 5′-GGATTTGCAGGTGTAGCTGT-3′ and the downstream primer sequence was 5′-GCTCCTGGGAAACAA GCAAC-3′. The actual length of the amplified fragment was 188 bp. Amplification was performed for three biological replicates at each time point, and *β-tubulin* gene of the *N*. *bombycis* served as the reference gene. The relative gene expression was calculated using delta-delta Ct.

### Analysis of the function of NbAQP

Primers were designed according to the sequence of the microsporidian aquaporin gene, *NbAQP* (GenBank accession number: KX655545). In order to exclude the effects of GFP reporter at the C terminus or N terminus of NbAQP, the pT7Ts-NbAQP, pT7Ts-eGFP, pT7Ts-NbAQP-eGFP, and pT7Ts-eGFP-NbAQP expression vectors were utilized. All of the constructed expression vectors were linearized by restriction enzyme digestion with *Sac* Ⅰ. In vitro transcription reactions were carried out according to the instructions outlined by manufacturer of the mMESSAGE mMACHINE T7 Kit (Ambion™). The resultant products were purified by phenol/chloroform extraction, and ethanol precipitation was used to recover cRNA after transcription.

Stage V and VI oocytes of *Xenopus laevis* were selected for injection. Each oocyte was injected with 40 ng of cRNA. In addition, two separate control groups of oocytes were either left uninjected or injected with an equal volume of DEPC water. Each group comprised of 50 oocytes. The oocytes were cultured in modified Barth's solution (88 mM NaCl, 1 mM KCl, 2.4 mM NaHCO_3_, 10 mM Hepes-NaOH, 0.33 mM Ca(NO_3_)_2_, 0.41 mM CaCl_2_, 0.82 mM MgSO_4_, pH 7.4, and 200 mOsm/kg, 50 U/mL penicillin, and 50 mg/mL streptomycin). After incubation for 2–3 days at 18°C, 10 oocytes exhibiting strong fluorescence intensity were selected under a fluorescence microscope and transferred to the same solution diluted fivefold with distilled water. Following transfer, the cells were placed under an inverted microscope and photographed every 30 s. Changes in oocyte volume were mapped for a total of 5 min and the water permeability (Pf) was calculated using the following formula:

Pf = V_0_[d(V/V_0_)/dt]/[S×Vw(Osm_in_-Osm_out_)], where V_0_ is the initial volume of oocytes, S is the initial surface area of oocytes, Vw is the partial molar volume of water molecules (18 cm^3^/mol), V is the volume of oocyte expansion and d(V/V_0_)/dt is the rate of change in oocyte volume over time [18].

### Rate of inhibition of antibody blocking on spore germination

A total of 20 μL of Percoll-purified spore suspension at a concentration of 2 × 10^7^ cells/mL was added to a sterile Eppendorf tube. Next, 100 μL of the antibody (at a concentration of 100 μg/mL) was added to the same tube and the tube was mixed at 30°C for 1 h; this served as the treatment group. The control group was given 100 μL antibody dilution and the blank group 100 μL of PBS. The control group and the blank group were also given 20 μL of an identical concentration of a spore suspension. These solutions were then thoroughly mixed and incubated at 30°C for 1 h. The incubation medium and the blank group were supplemented with 400 μL of germination medium, and the associated mixtures were thoroughly mixed and cultured at 30°C and 200 rpm for 1 h. A total of 400 μL of PBS was added to the control group mixture and the resultant suspension was incubated at 200 rpm in a 30°C shaker for 1 h. Next, 200 μL of culture media were obtained from each group and the OD value of the media was measured at a wavelength of 625 nm using a spectrophotometer. This experiment was performed 10 times and the data were recorded. After determining OD value, the spore germination rate and the inhibition rate of spore germination were calculated using the following formula:

Spore germination rate = (Control group OD625 − Test group OD625) / Test group OD625 * 100% Germination inhibition rate = (Blank group germination rate − Treatment group germination rate) / Treatment group germination rate * 100%

## Results

### Sequence characterization

**Fig 1:**
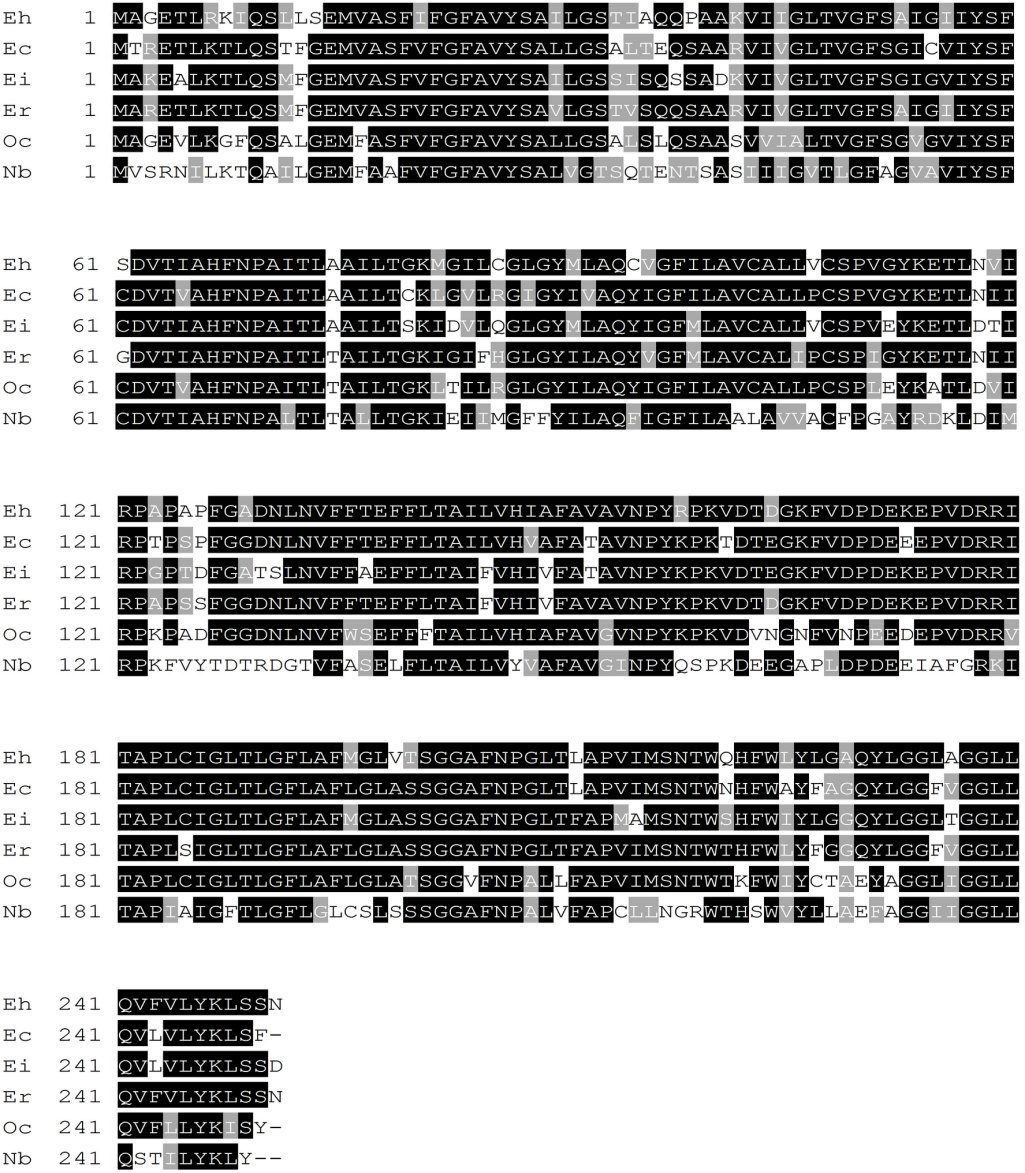
Amino acid sequence alignment of NbAQP and AQPs from other microsporidia. Eh: *Encephalitozoon hellem* (XP_003887579.1) homology with NbAQP was 51%; Ec: *Encephalitozoon cuniculi* (NP_586002.1) homology with NbAQP was 54%; Ei: *Encephalitozoon intestinalis* (XP_003073192.1) homology with NbAQP was 52%; Er: *Encephalitozoon romaleae* (XP_009264817.1) homology with NbAQP was 52%; Oc: *Ordospora colligata* (XP_014563378.1) homology with NbAQP was 56%; Nb: *Nosema bombycis* (API70721).

**Fig 2:**
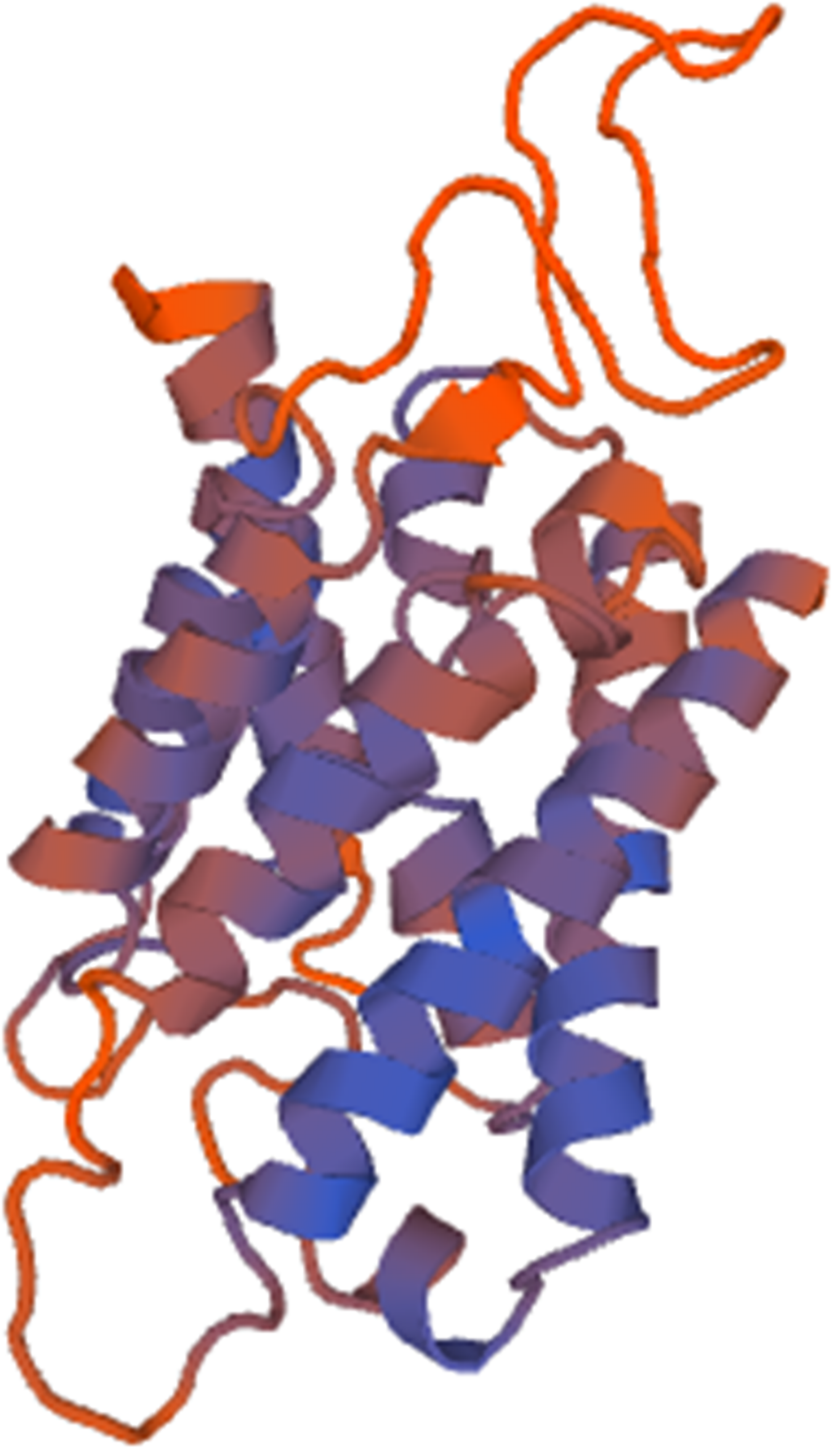
Tertiary structure prediction of NbAQP.

**Fig 3:**
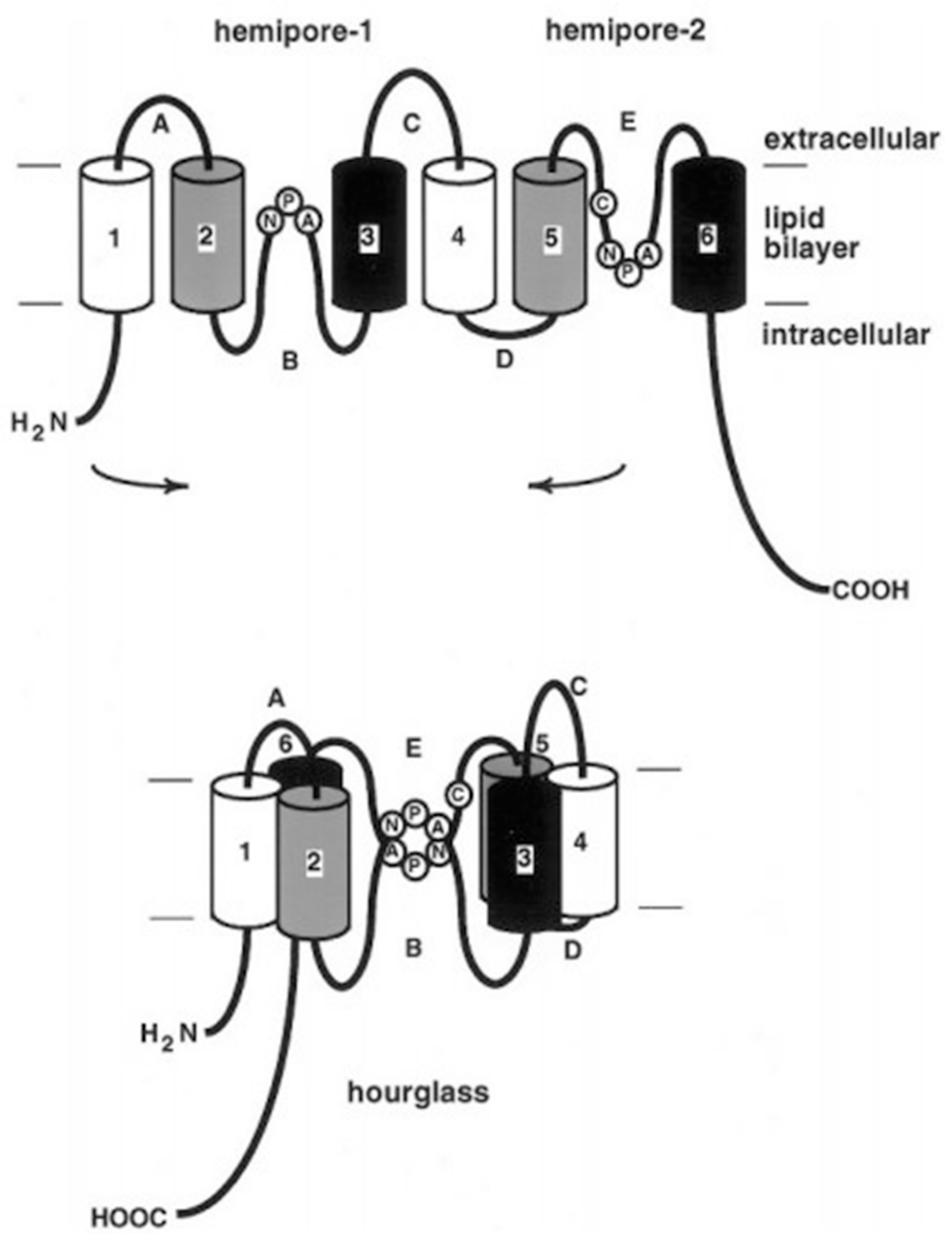
Schematic diagram of aquaporin structure.

**Fig 4:**
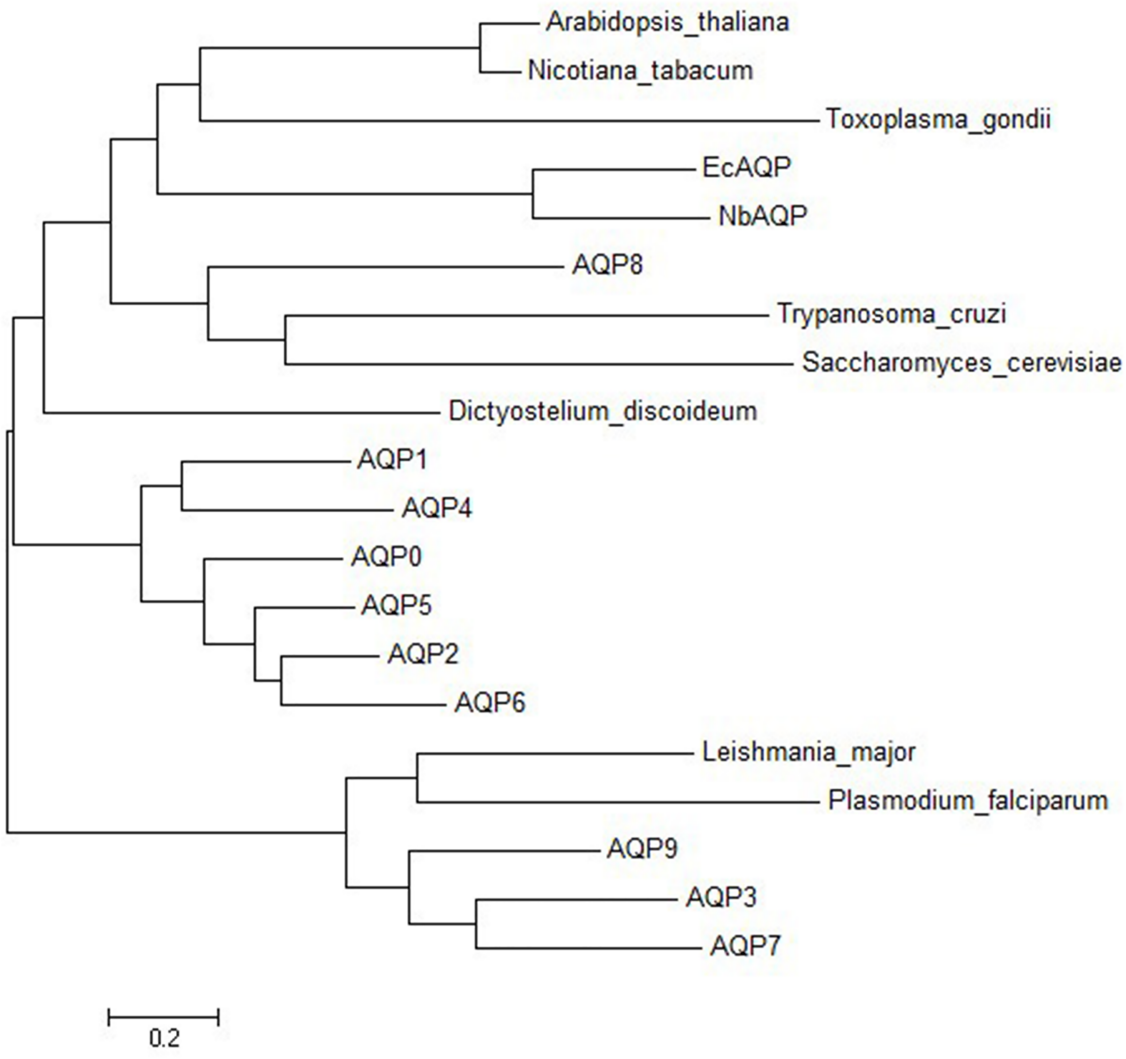
The phylogenetic tree of the aquaporins including NbAQP. Phylogenetic tree of NbAQP and other homologous protein sequences constructed using the Maximum Composite Likelihood method within the package MEGA 6.0. *Encephalitozoon cuniculi* (NP_586002), *Dictyostelium discoideum* (BAA85158), human AQPs 0–9 (NP_036196, NP_932766, NP_000477, NP_004916, P55087, NP_001642, Q13520, NP_001161, O94778, NP_066190, respectively), plant aquaporins of *Arabidopsis thaliana* (P25818) and *Nicotiana tabacum* (CAA69353), parasitic protist aquaporins of *Leishmania major* (AAS73184), *Plasmodium falciparum* (CAC88373), *Toxoplasma gondii* (CAE46485) and *Trypanosoma cruzi* (AAM76680), and AQP 2 of *Saccharomyces cerevisiae* (AAD10058). Human AQP3, AQP7, AQP9, and the aquaporins of *Leishmania major*, *Plasmodium falciparum* and *Toxoplasma gondii* are aquaglyceroporins.

An ORF finder was used to predict the nucleotide and amino acid sequences, respectively. The open reading frame of *NbAQP* was 750 bp long and encodes a protein of 249 amino acids. The predicted molecular weight of NbAQP was 26.66 kDa and the isoelectric point was 5.12. No signal peptide sequence was observed. SMART software was subsequently utilized to identify characteristic motifs in the sequence. We observed that the sequence contained a distinct transmembrane domain. A subsequent transmembrane domain analysis of the amino acid sequence of NbAQP was carried out using the TMpred program. The results indicated that the protein sequence contains six transmembrane domains, which are located at the 12–31, 433–66, 87–113, 133–154, 184–204 and 224–246 amino acid positions. The amino acid sequence encoded by *NbAQP* was compared to that of five other microsporidian species from the NCBI database. The homology between the six sequences was greater than 50%, and the homology with EcAQP was 54% (Fig 1). In accordance with analysis using the SWISS-MODEL server, the tertiary structure of NbAQP was predicted to contain a conserved transmembrane domain that is normally associated with the major intrinsic protein (MIP) family of transport protein channels. Each of the homologous aquaporins contains six transmembrane domainslinked to one another by five loop structures. The A, C, and E loops are located on the outside of the membrane, and the B and D rings are located on the inside of the membrane. The B and D loops contain two highly conserved NPA (Asn-Pro-Ala) motifs that are commonly found in aquaporins, forming a narrow channel that gives rise to an hourglass structure (Fig 2). The presence of these conserved structures may be an important factor in the specificity of substrate selection (Fig 3). A phylogenetic tree displaying aquaporins, including NbAQP, was generated using MEGA6 (Fig 4). NbAQP and EcAQP clustered together in the resultant phylogenetic tree, suggesting that these two proteins are functionally similar and share a common origin.

### Expression of NbAQP and antibody preparation

**Fig 5:**
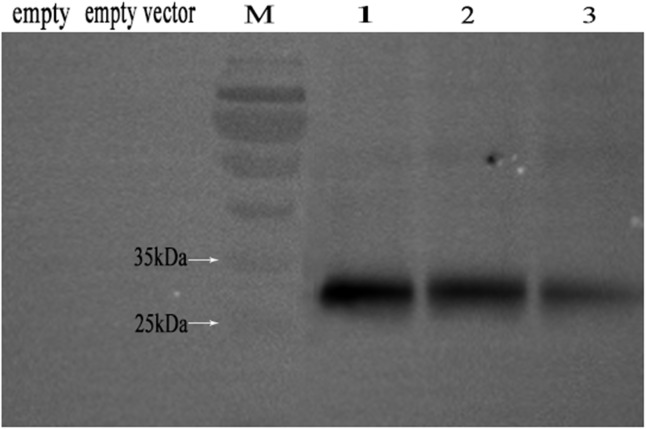
Western blotting analysis of His-NbAQP fusion protein. Lane M: Marker; Lane 1: His-NbAQP fusion protein after 15 h of induction; Lane 2: His-NbAQP fusion protein after 20 h induction; Lane 3: His-NbAQP fusion protein after 24 h induction.

The results of Western blotting of His-NbAQP fusion protein revealed a 31-kDa band at 15 h, 20 h, and 24 h after β-galactose induction, and no bands were observed for the empty strain *S*. *cerevisiae* and empty vector pYES2-NTC samples (Fig 5). We therefore concluded that NbAQP was successfully expressed in the yeast expression system. We also observed that protein expression varied at the different points in time after induction.

**Fig 6:**
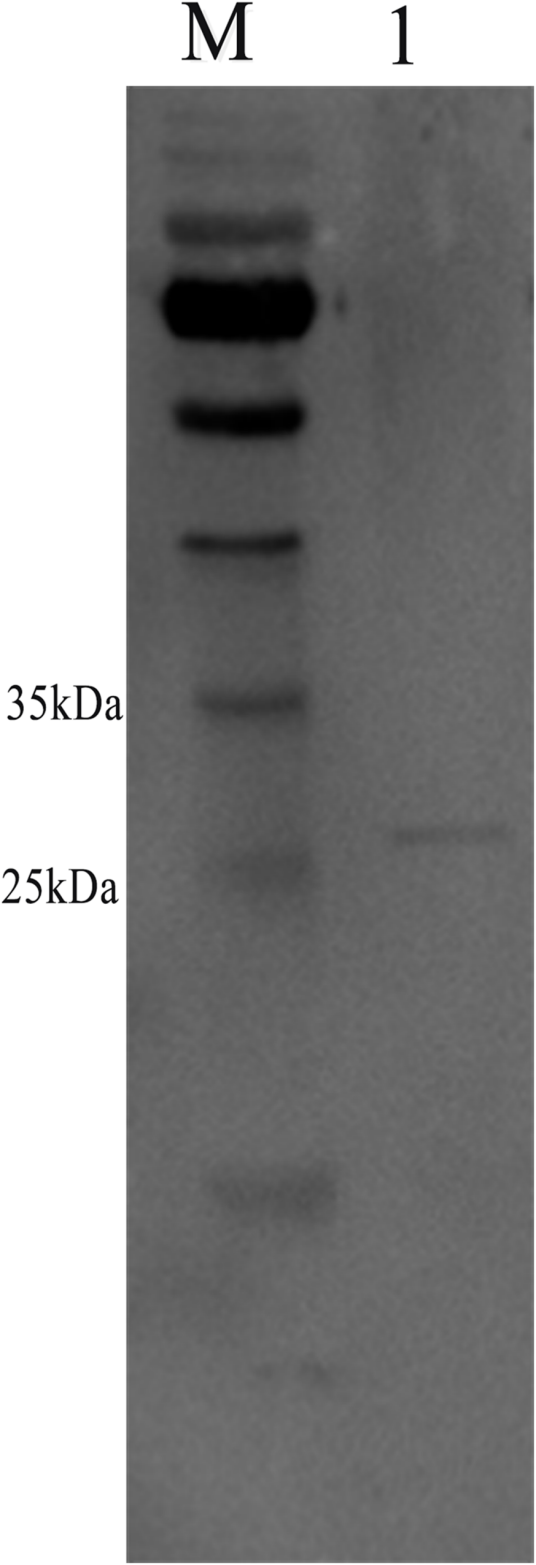
Specificity analysis of NbAQP antibody. Lane M: Prestained marker. Lane 1: Total protein from *N*. *bombycis* containing NbAQP.

The specificity of the polyclonal antibody was tested by Western blotting, where the NbAQP protein polyclonal antibody served as the primary antibody. The results indicated that when the NbAQP protein polyclonal antibody served as a primary antibody and HRP-labeled goat anti-rabbit antibody as a secondary antibody, a protein of approximately 27 kDa was detected in the total protein extracted from spores. This band size is consistent with the predicted molecular weight of the NbAQP protein (Fig 6).

### Localization of NbAQP

**Fig 7:**
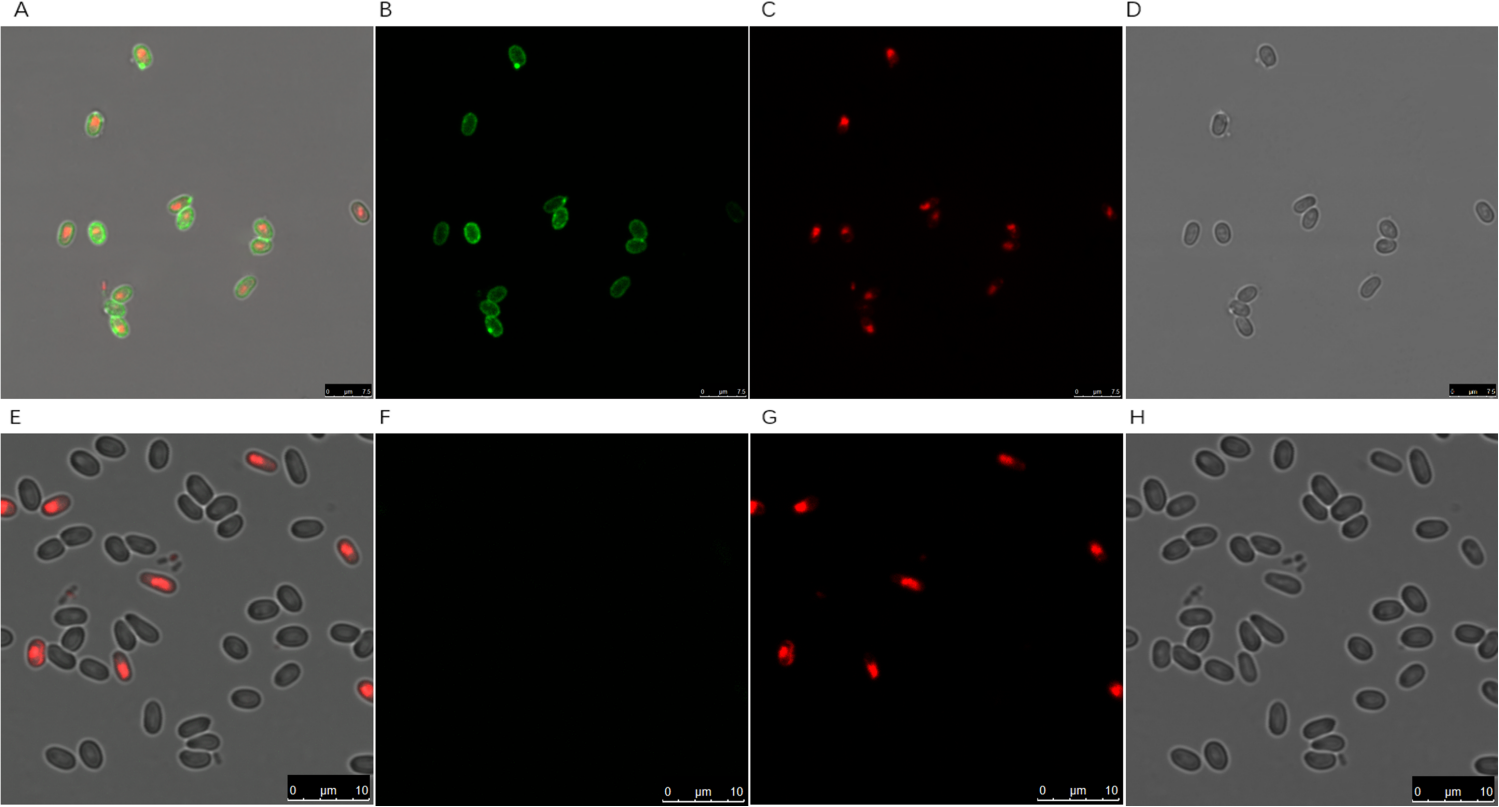
Indirect immunofluorescence. (A): Combination of (B) and (C). (E): Combination of F and G. (B): *N*. *bombycis* following incubation with primary antibody and secondary antibody. (C) and (G): *N*. *bombycis* cell nucleus stained with PI. (D) and (H): *N*. *bombycis* visualized using phase contrast microscope. (F): *N*. *bombycis* following incubation with negative serum and secondary antibody.

Following indirect immunofluorescence with the polyclonal antibody (Fig 7), an intense green fluorescent signal was observed in the *N*. *bombycis* cell wall, no fluorescence was observed for the negative control, suggesting that the *N*. *bombycis* aquaporin may be located in the spore wall.

**Fig 8:**
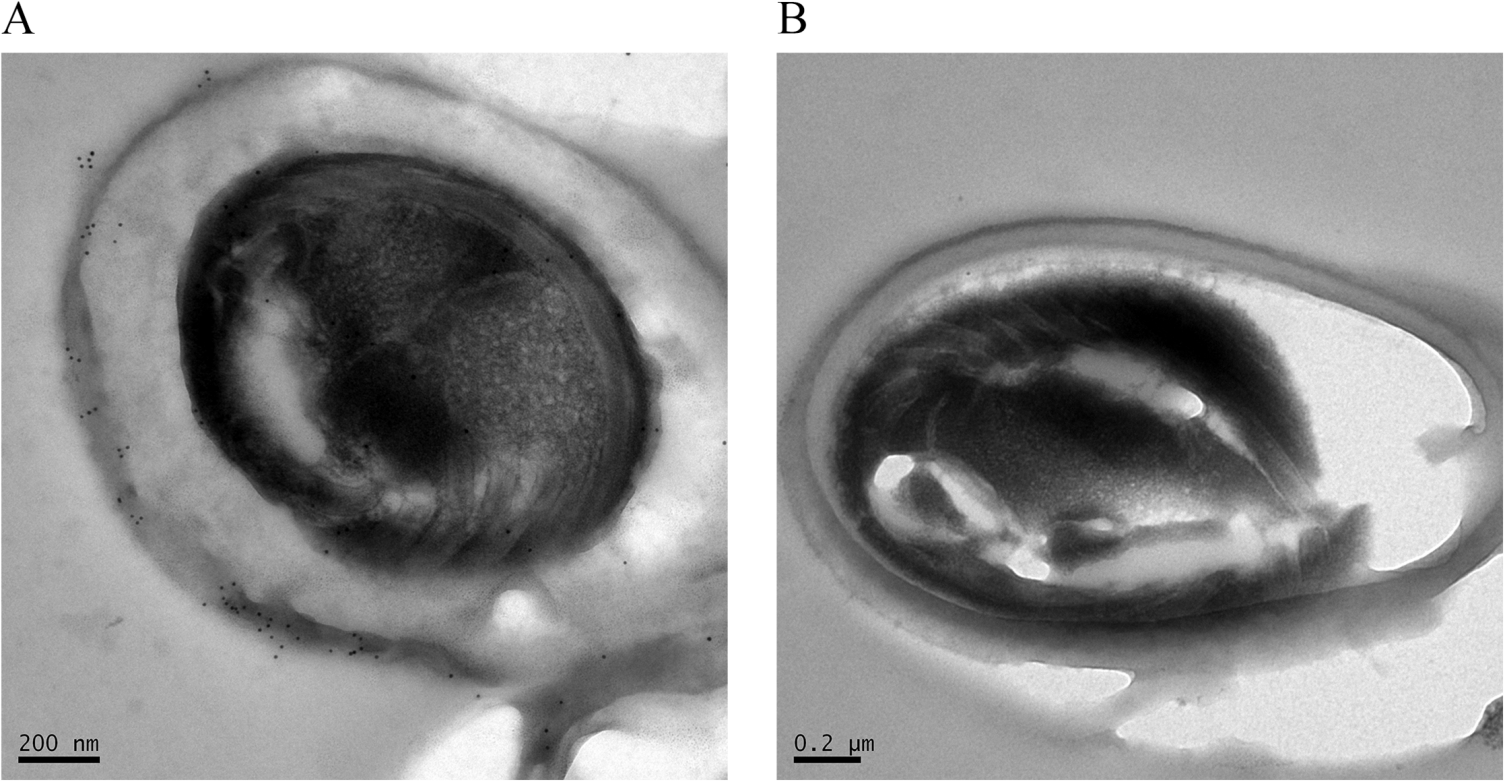
Immunogold electron microscopy. Under immunogold electron microscopy, the *N*. *bombycis* spore wall appeared to be thick and relatively rough with non-uniform electron density (Fig 8). Colloidal gold particles were loosely distributed in the spore wall, and there was also limited localization of colloidal gold particles in the plasma membrane (Fig 8A). Conversely, no colloidal gold particles were detected in the spore walls or cytoplasm of the control group (Fig 8B).

Under immunogold electron microscopy, the *N*. *bombycis* spore wall appeared to be thick and relatively rough with non-uniform electron density (Fig 8). The proteins identified using the polyclonal antibody were predominantly located in the spore wall. Colloidal gold particles were loosely distributed in the spore wall, and there was also limited localization of colloidal gold particles in the plasma membrane (Fig 8A). Conversely, no colloidal gold particles were detected in the spore walls or cytoplasm of the control group (Fig 8B).

### Real-time fluorescence quantitative PCR

**Fig 9:**
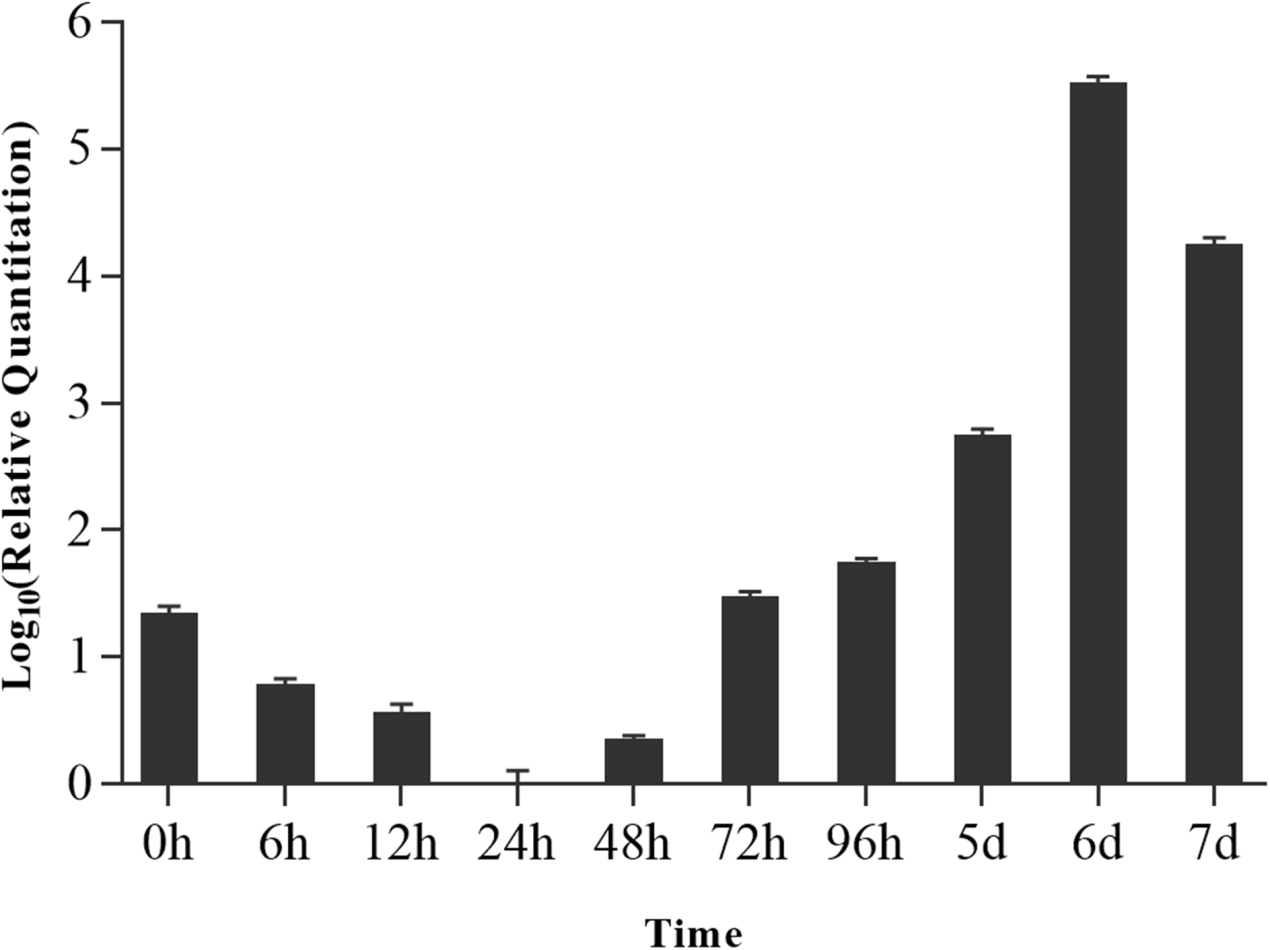
The relative expression level of NbAQP gene in the midgut of silkworm larvae at different time points after infection with *N*. *bombycis*. The y-axis indicates the relative expression level of NbAQP gene, and the x-axis indicates the time. Vertical bars represent the mean ± SE (n = 3).

After infecting the silkworm with *N*. *bombycis*, the relative expression level of *NbAQP* mRNA changed markedly at different points in time (Fig 9). The expression of *NbAQP* was detected in the larval midgut within 7 days after infection of the silkworm, and there is a high expression at 0 h after inoculation. The relative expression of *NbAQP* was down-regulated significantly between 0 h and 24 h, significantly increased from 24 h, and reached the highest level of expression at the 6th day post-infection (Fig 9).

### Water channel function of NbAQP

**Fig 10:**
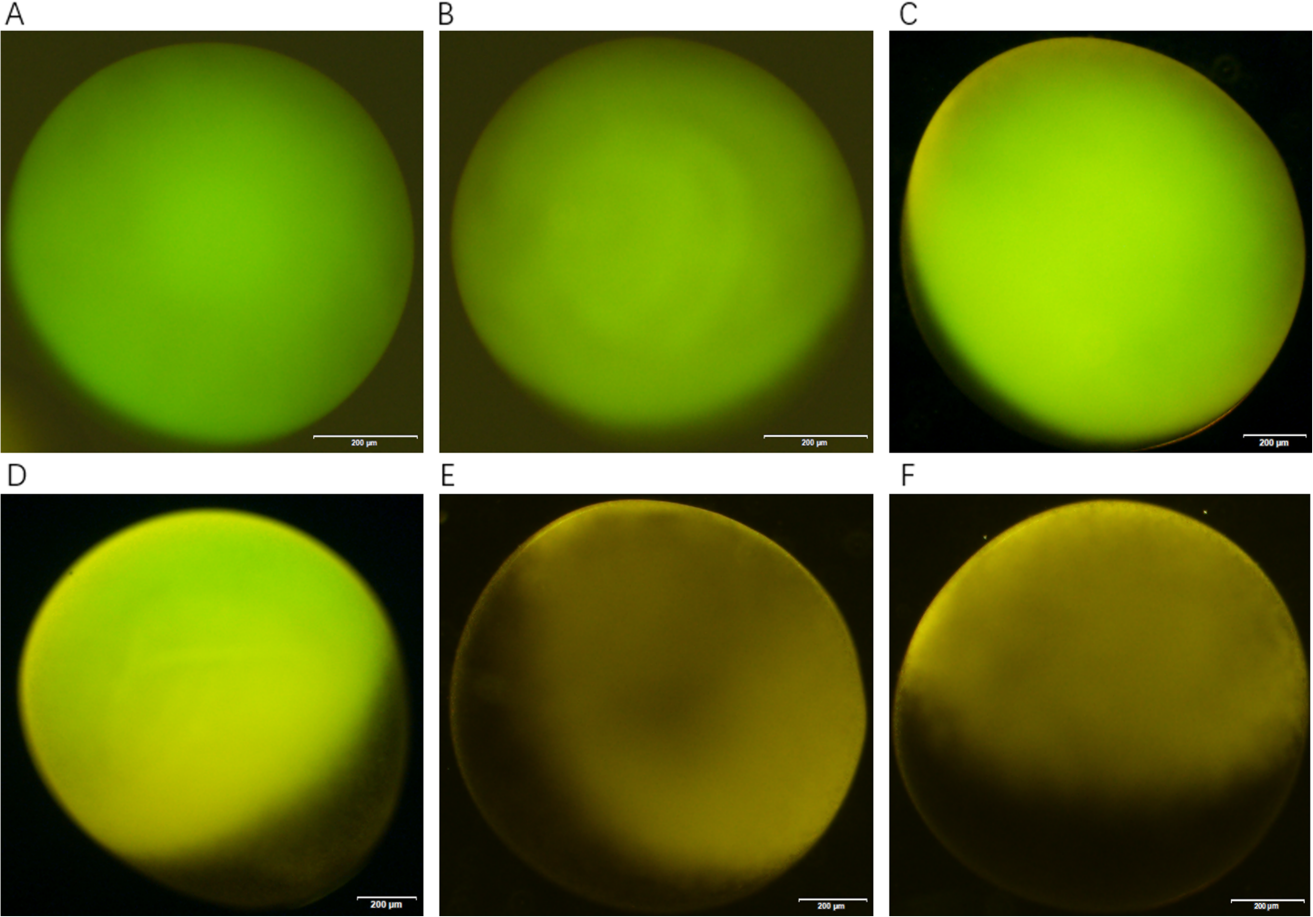
Resultant oocytes after expression of the fusion protein GFP and NbAQP. (A) Cells injected with T7Ts-EGFP; (B) cells injected with T7Ts-EGFP +T7Ts-NbAQP; (C) cells injected with T7Ts-EGFP-NbAQP; (D) cells injected with T7Ts-NbAQP-EGFP; (E) non-injected cells; (F) cells injected with DEPC water.

**Fig 11:**
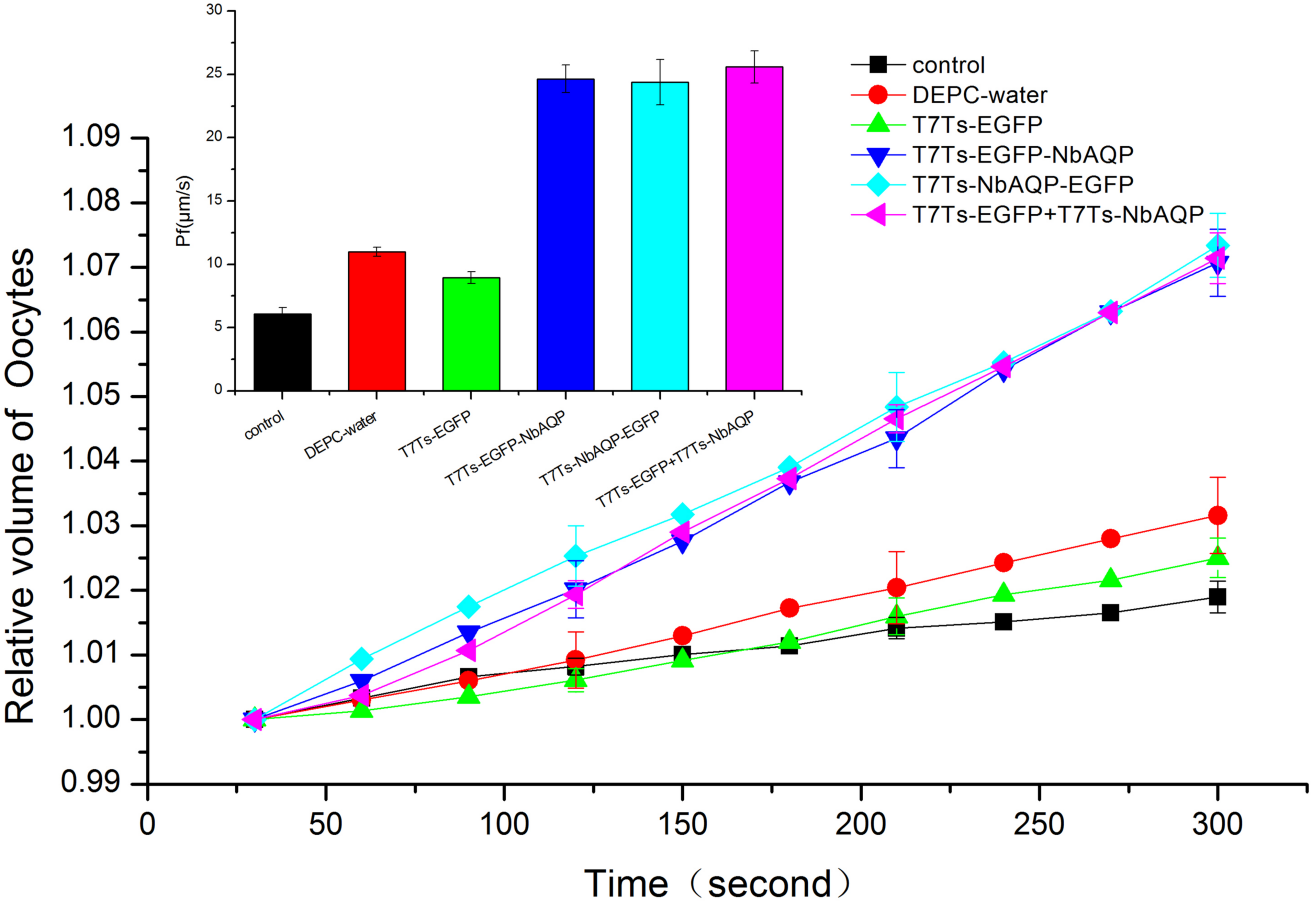
Expansion experiment after *NbAQP* injection into *Xenopus laevis* oocytes. At 5 min after injection with pT7Ts-NbAQP-eGFP, pT7Ts-eGFP-NbAQP and pT7Ts-NbAQP + pT7Ts-eGFP.(the expansion rate was 7.1% ± 0.52%, 7.3% ± 0.49% and 7.1% ± 0.39%, respectively; the Pf was 24.7 μm/s, 24.4 μm/s and 25.6 μm/s, respectively, (*p* < 0.05)) were more than those groups injected with DEPC water, pT7Ts-eGFP and non-injected (the expansion rate was 1.9% ± 0.24%, 3.2% ± 0.5%, and 2.5% ± 0.3%, respectively; the Pf was 6.1 μm/s, 11 μm/s and 8.9 μm/s, respectively (p < 0.05)) (n = 10). (Vertical bars represent the mean ± SE; for clarity, only three error bars are displayed.)

A total of 40 ng of NbAQP cRNA or the same volume of DEPC water was injected into *Xenopus laevis* oocytes using the heterologous expression system. The resultant oocytes were observed after 3 d using an inverted microscope (Fig 10). Oocyte volume expansion rate and cell permeate permeability were calculated for the observed oocytes (Fig 11). In these experiments, the oocytes injected with pT7Ts-NbAQP-eGFP, pT7Ts-eGFP-NbAQP and pT7Ts-NbAQP + pT7Ts-eGFP (the expansion rate were 7.1% ± 0.52%, 7.3% ± 0.49% and 7.1% ± 0.39%, respectively; the value of Pf were 24.7 μm/s, 24.4 μm/s and 25.6 μm/s, respectively, (p < 0.05)) were larger than those groups injected with DEPC water, pT7Ts-eGFP and non-injected (the expansion rate was 1.9% ± 0.24%, 3.2% ± 0.5% and 2.5% ± 0.3%, respectively; the Pf was 6.1 μm/s, 11 μm/s and 8.9 μm/s, respectively (*p* < 0.05)). The results indicated that, compared with the control group, the injection of oocytes with NbAQP resulted in rapid expansion.

### Inhibition of spore germination after antibody blocking

**Fig 12:**
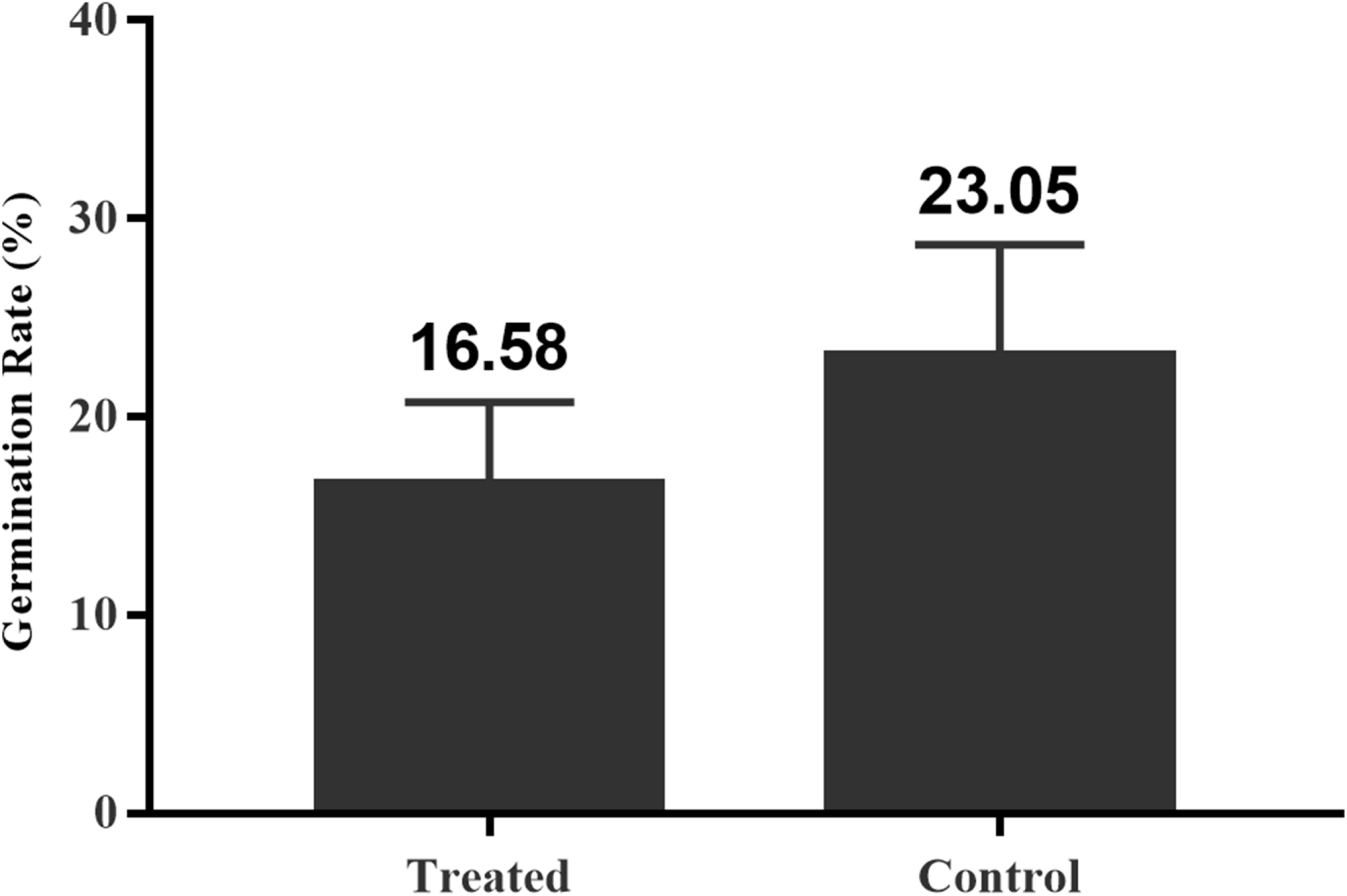
Spore germination rate. The experimental results showed that the average germination rate of spores was 23.05% in the blank control group and 16.58% in the group that was exposed to the blocking antibody. Vertical bars represent the mean ± SE (n = 10).

In the antibody blocking test, the OD values of the control group, treatment group, and blank group were determined using an ultraviolet-visible full-wavelength spectrophotometer (at a wavelength of 625 nm). The OD value of each group was calculated based on the OD value of the control group. The germination rate of the spores exposed to the different treatment conditions was calculated using to the formula outlined in Section 2.6 Inhibition rate of antibody blocking on spore germination (Fig 12). The inhibition rate of antibody blocking on spore germination was calculated. The experiment was performed 10 times and the results showed that the average germination rate of spores was 23.05% in the blank control group and 16.58% in the group that was exposed to the blocking antibody for 1 h. The difference in the germination rate between the two groups was significant (*P* < 0.05). The inhibition rate in relation to spore germination following antibody blocking was approximately 28%.

## Discussion

For the first time, this study demonstrated that aquaporins is present in *N*.*bombycis* and function as water channel. Aquaporins play a key role in the natural biological activities of many different organisms. Members of the aquaporin family are found in archaea, eubacteria and eukaryotes [19], including fungi, animals, and plants. They perform an astonishing variety of physiological functions and are easily identified by sequence similarity across all kingdoms of life [20–22]. There are three kinds aquaporins in mammals: orthodox AQP, which can permeable to water, aquaglyceroporin, which can permeable to glycerol and other small solutes, and non-orthodox (AQP11 and 12) [23]. Aquaporins are thought to exist natively as a homotetramers, with each 26–34 kDa monomer [24]. In this study, sequence analysis revealed that the *N*. *bombycis* aquaporin has a series of characteristics in common with the rest of aquaporin family. The expected molecular weight of the NbAQP protein is 26.66 kDa, within the range for an AQP monomer. Phylogenetic tree analysis revealed that NbAQP and EcAQP share a common origin and similar functional characteristics. This strongly suggests that NbAQP is a member of the AQP protein family. Indirect immunofluorescence assay and immunogold electron microscopy showed that NbAQP is mainly located on spore walls. This is consistent with the predicted result that the NbAQP is a transmembrane protein and has six transmembrane domains (Fig 2). Many plant aquaporins have been found to perform water channel functions, and most of these proteins are localized at the plasma membrane [21, 25]. The current study revealed that the aquaporin of *N*. *bombycis* might be a spore surface protein, and it most likely participates in spores’ germination.

Although the expansion rate of the oocytes treated with NbAQP was significantly higher than that of the control group, the Pf for NbAQP was much lower than those observed in fungi [26], *Plasmodium falciparum* (276 μm/s) [27], *Trypanosoma cruzi* (32 μm/s) [28], *Encephalitozoon cuniculi* (*EcAQP*) (87 μm/s) [29], and human aquiporins, the Pf values for human AQP3, 2 and 5 were 80, 100, and 100 μm/s, respectively [30]. The reasons for these differences remain to be elucidated. Several regulatory mechanisms have been shown to modulate the activity of AQPs. However, due to variations in expression levels caused by non-standardized injection of cRNA and varying translational and post-translational processing efficiencies, the comparisons that were drawn during these analyses were not strictly quantitative [29]. Previous AQPs have one or two short, highly conserved sequences called NPA (asparagine-proline-alanine) boxes. Each one of the NPA boxes has a span of relatively hydrophobic amino acids. They form loops directed into the membrane, which form a pore, as revealed by three-dimensional structure analyses of AQP [31]. In this paper, NbAQP was predicted to contain conserved transmembrane domain and two NPA boxes, which is usually found in most AQPs. However, there is still no enough evidence to predicate the NbAQP protein belong to what kind of AQP, so further research into solute transport and other function of the NbAQP is essential.

The germination of *N*. *bombycis* spore requires appropriate conditions with respect to pH, osmotic pressure, and changes in temperature. Changes in external conditions can lead to changes in the permeability of the spore wall, causing increase in osmotic pressure within the spore. Spore formation is a very important stage in the fungal life cycle that allowa the organism to survive adverse conditions [32]. Spores are capable of staying in a dormant state until they encounter favorable environmental conditions. As part of this analysis, we observed that NbAQP can promote rapid water penetration into the oocytes, but the water permeability is very low after expression of NbAQP. It is likely that NbAQP has other significant functions. Previous reports have suggested that the aquaporins might also be involved sensing and response to environmental signals [19]. Spores from different fungal species have different requirements with respect to the initiation of germination [33].The regulation of water uptake during germination is an integral part of the fungal life cycle [34].

However, the regulatory mechanisms that underpin aquaporin activity are not clear. Monovalent ions are essential in vitro stimuli for the germination of *N*. *bombycis* [35], *Nosema fumiferanae* [36], *Pleistophora anguillarum* [37], and *Vavraia culicis* [3]. It has been reported that the pH of the external environment surrounding the spores is an important factor in the stimulation of spore germination [19]. Different regulatory mechanisms have been shown to have direct effects on the structure of aquaporins [38]. In terms of pH regulation, histidine has been reported to be protonated in acidic environments. This leads to what is known as the gating mechanism [39, 40]. When the microsporidia infect the host cell, spores must first germinate and then inject the infective sporoplasm into cytoplasm of the host cell through the polar filament. So, the germination of *N*.*bombycis* need suitable environment, this means exist mechanism which can open the channel of NbAQP to permeate water into intracellular. The regulation of aquaporin expression in *N*. *bombycis* is of great significance in the elucidation of regulatory mechanisms associated with microsporidian germination. Thus, additional experiments are necessary to determine the mechanisms underlying the regulation of aquaporins in *Nosema bombycis*, which may help elucidate the mechanisms underlying spore germination in *Nosema bombycis*.
